# Toll-like receptor 2-mediated NF-kappa B pathway activation in ocular surface epithelial cells

**DOI:** 10.1186/s40662-017-0082-x

**Published:** 2017-07-11

**Authors:** Aihua Hou, Min Qi Tin, Louis Tong

**Affiliations:** 1 Ocular Surface Research Group, Singapore Eye Research Institute, Singapore, Singapore; 2 Duke-NUS Graduate Medical School, Singapore, Singapore; 3 Singapore National Eye Center, Singapore, Singapore; 4 Yong Loo Lin School of Medicine, National University of Singapore, Singapore, Singapore

**Keywords:** TLR2, NF-κB pathway, Activation, Ocular surface cells

## Abstract

**Background:**

Gram-positive bacteria stimulate Toll-like receptor (TLR) 2 and then activate the pro-inflammatory nuclear factor-kappa B (NF-κB) pathway. As the human ocular surface is heavily colonised by gram-positive cocci bacteria, a balance of activation/repression of NF-κB target genes is essential to avoid uncontrolled infection or autoimmune-related inflammation. It is advantageous to test NF-κB targeting molecules in an ocular surface culture system that allows assessment of temporal NF-κB activation in a longitudinal fashion without destruction of cells. Such initial testing under standardised conditions should reduce the number of molecules that progress to further evaluation in animal models. This study aims to establish an in-vitro cell culture system to assess NF-κB activation in the context of ocular surface cells.

**Methods:**

NF-κB activity was evaluated through a secretory alkaline phosphatase reporter assay (SEAP). Immunoblots and immunofluorescence were used to examine IκBα phosphorylation and p65/p50 nuclear localization. Monocyte chemoattractant protein-1 (MCP-1) transcripts were evaluated by real time PCR and protein levels were measured by ELISA.

**Results:**

NF-κB activity in HCE-T cells treated with TLR2 activator Pam3CSK4 was higher than control cells at both 6 and 24 h. Pam3CSK4-stimulated NF-κB activation was inhibited by IκK inhibitors, Wedelolactone and BMS-345541. In Pam3CSK4 treated cells, active NF-κB subunits p50 and p65 increased in cell nuclear fractions as early as 1.5 h. Although the level of total IκB-α remained constant, phospho-IκB-α increased with treatment over time. In the culture media of Pam3CSK4-stimulated cells, MCP-1 protein level was increased, which was suppressed in the presence of IκK inhibitors.

**Conclusion:**

NF-κB pathway can be activated by the TLR2 ligand and inhibited by IκK inhibitors in the ocular surface cell culture system. This cell culture system may be used to evaluate TLR-related innate defences in ocular surface diseases.

## Background

The ocular surface is constantly exposed to the colonization of various commensal microbes, playing an important role in defence against microbes and other inflammatory insults [1, 2]. The nuclear factor kappa (NFκ) B transcription pathway is the central regulator of ocular surface inflammation and disease [3–7]. Toll-like receptors (TLRs) are a family of transmembrane receptors that recognize microbial pathogens and trigger early innate immune responses leading to inflammation in mammalian cells [8–10]. TLRs present on the membrane of ocular surface cells can recognize and bind to a variety of microbial components including bacterial lipopeptides, lipopolysaccharide (LPS), flagellin, viral dsRNA, ssRNA and other ligands [11–13]. On activation of TLR, subsequent signalling of the cells can activate the master kinase called the Ikappa-B kinase (IκK), which phosphorylates the Ikappa-B alpha (IκBα) subunit of NF-κB. The phosphorylated IκBα undergoes degradation, releasing the p50 and p65 subunits of NF-κB, which translocate to the cellular nuclei to bind to the promoter of target genes [3, 8, 14]. Through up-regulation, and less often, down-regulation of target genes, this signalling pathway has a major influence on the local immune defence and may be critical in control of ocular infections.

The regulation of NF-κB is highly context- and tissue-dependent, so it is important to define the triggers and targets of this pathway in healthy ocular surface and in disease. TLR2 is reported to function as a Gram-positive bacteria sensor in the cornea [12]. In Gram-positive bacterial infections, pro-inflammatory mediators such as TNF-alpha, interleukin (IL)-6, IL-8 and the adhesion molecule ICAM-1 were secreted by human cornea epithelial cells through activation of NF-κB [15–17]. The propagation of inflammation by these molecules can increase the severity of infection. On the other hand, NF-κB-mediated expression of anti-microbial molecules human defensin (hBD)-2 and homeostatic molecules manganese superoxide dismutase may help to control the pathogenicity of microbes or increase defences against oxidative stress [15, 18]. It is therefore important to understand how NF-κB is finely tuned in health and disease involving ocular surface cells, so that appropriate therapeutic strategies can be implemented to protect the eye against ocular infections [8].

We aim to describe the activation of NF-κB in an in-vitro system of ocular surface cells using promoter assay and other approaches, induced by the presence of a TLR2 ligand.

## Materials and methods

### Cells

Human SV-40 immortalized corneal epithelial cell line (HCE-T) cell line was obtained from the Riken Cell Bank (RCB2280) [19]. Cells were cultured with DMEM/F-12 (Life Technologies, CA, USA) medium supplemented with 5% foetal bovine serum (FBS) (Life Technologies, CA, USA) and maintained in a 37 °C incubator with 5% CO_2_. Medium was changed every 2 days. Cells with passage no. 69–73 were used in this study.

### Transfection, IκK inhibitor treatment and Pam3CSK4 stimulation

HCE-T cells were cultured to 90% confluent in 6-well plates and changed to fresh medium before transfection. Six microliters of Fugene 6 Transfection Reagent (Promega, Madison, USA) and 200 μl of Epilife medium (Life Technologies, CA, USA) were mixed and incubated at room temperature for 5 min. Subsequently, vectors (2 μg) pSEAP-basic or pSEAP-NF-κB (Clontech Laboratories, Mountain View, CA) were added to the mixture and incubated at room temperature for 15 min. The mixture was then added to the cells and incubated in a 37 °C incubator with 5% CO_2_ overnight.

**Fig. 1:**
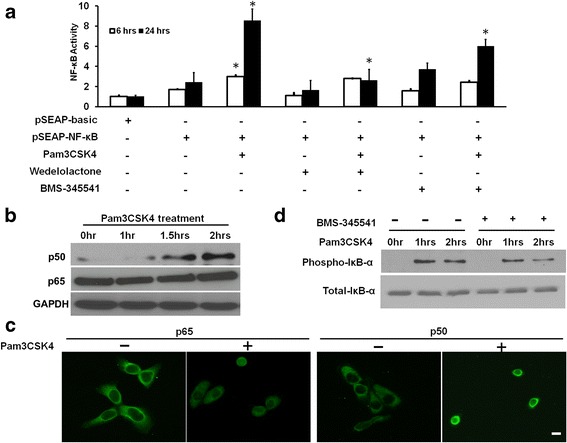
**a** NF-κB transcriptional activity assay. HCET cells were transfected with pSEAP-basic or pSEAP-NF-κB vector, treated with Pam3CSK4 (a TLR2 ligand) for 6 and 24 h with or without the presence of IκK inhibitors Wedelolactone and BMS-345541. Height of each bar represents mean of two independent experiments and is normalized to negative control (pSEAP-basic). The error bars represent standard error of the means. **b** HCE-T cells were treated with Pam3CSK4 for various time intervals and nuclear fraction of treated cells were immunoblotted with antibodies against p50 and p65. **c** HCE-T cells seeded in chamber slides were treated with Pam3CSK4 for 4 h and immunofluorescent staining was performed using primary antibodies against p65 and p50, and Alexa Fluor 488 conjugated secondary antibody (green). Representative images are shown. Scale bar = 50 μm. **d**. HCE-T cells were treated with Pam3CSK4 with or without IκK inhibitors for various time intervals and total cell lysate was immunoblotted with antibodies against phospho-IκB-α and total IκB-α. GAPDH was used as the endogenous loading control. *: *P* < 0.05

IκK inhibitor Wedelolactone (Calbiochem, San Diego, CA) or BMS-345541 (Calbiochem, San Diego, CA) was added to select wells, which were transfected with pSEAP-NF-κB at a concentration of 10 μM or 5 μM, respectively. Pam3CSK4 is a well-established TLR2 specific ligand [20]. Depending on the experiment group assignment, Pam3CSK4 was added to selected wells at a final concentration of 250 ng/mL an hour later. The assignment of cell transfection and treatment groups is depicted in Fig. 1a.

### NF-κB transcriptional activity detected by secreted alkaline phosphatase (SEAP) assay

NF-κB transcriptional activity was detected using the Great EscAPe SEAP detection kit (Clontech Laboratories, Mountain View, CA) following the manufacturer’s instruction. Briefly, culture media (400 μl) were collected from cells transfected with different pSEAP vectors with or without Pam3CSK4 stimulation and IκK inhibitors treatment. The culture media were centrifuged at 12,000 rpm for 10 s before adding to a 96-well black walled plate (12 μl per well). Fresh media was added (48 μl per well) to the culture medium. Serial dilutions of the positive control alkaline phosphatase were also added to the same 96-well plate. The plate was sealed with adhesive aluminium foil, incubated at 65 °C for 30 min, and then cooled on ice for 2–3 min. After equilibrium to room temperature, 60 μl of assay buffer was added to each well and the plate was incubated for 5 min at room temperature. Subsequently, 60 μl of substrate working solution was added and the plate was incubated at room temperature for 10 min. Chemiluminescence was measured by Tecan GENios (Tecan, Männedorf, Switzerland).

### Nuclear extraction

Nuclear extraction from HCE-T cells stimulated with Pam3CSK4 was performed using the nuclear extraction kit (Active Motif, Carlsbad, CA) following the manufacturer’s instruction. Briefly, HCE-T cells were stimulated with Pam3CSK4 (250 ng/ml) for 0, 1, 1.5 and 2 h in 100 mm culture plates, washed with 10 ml of cold PBS, and scrapped off from culture plates with 400 μl of cold PBS containing protease and phosphatase inhibitors (Roche Applied Science, Penzburg, Germany). Scrapped cells were transferred to pre-chilled tubes and centrifuged for 5 min at 500 rpm at 4 °C. Cell pellet was re-suspended in 500 μl of 1X hypotonic buffer and incubated on ice for 15 min. Twenty-five microliters of detergent was added to the cell pellet suspension and centrifuged for 30 s at 14,000 rpm at 4 °C. The pellet was re-suspended in 50 μl of complete lysis buffer and incubated for 30 min on ice with shaking at 150 rpm, and then centrifuged at 14,000 rpm for 10 min at 4 °C. The nuclear containing supernatant was transferred into pre-chilled 1.5 ml tubes and stored at −80 °C until use. Protein concentration was determined by BCA method following manufacturer’s instruction [21].

### Western Blot

Western blots were performed as described previously [22]. Briefly, protein samples (30 μg per lane) were separated in 12% SDS-PAGE gel and transferred to PVDF membranes. The membranes were then blocked with 5% BSA in TBS-Tween 20 (TBS-T) for 1 h at room temperature and incubated with primary antibodies for 2 h at room temperature. After that, the membranes were incubated with horseradish peroxidase conjugated secondary antibodies for 1 h at room temperature, washed with TBS-T for three times (5 min each time), incubated with SuperSignal West Pico chemiluminescent substrates (Pierce Biotechnology, Rockford, USA) and signals were visualized on X-ray films. Primary and secondary antibodies used in this study are listed in Table 1.

**Table 1:** Antibodies used in the study

Antibody	Species & Type	Manufacturer	Catalogue Number	Dilution factor
IκB-α	rabbit polyclonal	Santa Cruz	SC-371	1:1000
NF-κB p50	goat polyclonal	Santa Cruz	SC-1190	1:20 for IF, 1:1000 for WB
NF-κB p65	rabbit polyclonal	Santa Cruz	SC-109	1:20 for IF 1:1000 for WB
phosphor-IκB-α (Ser32/36) (5A5)	mouse monoclonal	Cell Signalling Technology	#9246	1:5000
Anti-Rabbit HRP conjugated secondary antibody	goat polyclonal	Santa Cruz	SC-2030	1:5000
Anti-mouse HRP conjugated secondary antibody	goat polyclonal	Santa Cruz	SC-2005	1:5000
Anti-goat HRP conjugated secondary antibody	rabbit polyclonal	Santa Cruz	SC-2768	1:5000
Anti-Rabbit Alexa Fluor 488 conjugated secondary antibody	goat polyclonal	Sigma-Aldrich	SAB4600234	1:400
Anti-goat Alexa Fluor 488 conjugated secondary antibody	donkey polyclonal	Abcam	Ab150129	1:1000

### Immunofluorescence staining

Immunofluorescent staining of nuclear translocation of NF-κB active subunits, p50 and p65, were performed according to a previous protocol [23]. In summary, cells seeded in chamber slides treated with or without Pam3CSK4 were fixed with 100% methanol for 10 min at room temperature, washed with PBS, permeated in PBS containing 0.15% Triton X-100 for 15 min, blocked with 4% BSA in PBS containing 0.1% Triton X-100 (Sigma) for 1 h, then incubated with primary antibodies at 4 °C overnight. After washing with PBS containing 0.1% Tween-20, cells were incubated with Alexa Fluor 488-conjugated secondary antibodies at room temperature for 45 min. Subsequently, chamber walls were removed and slides were mounted with VectaShield mounting medium with DAPI (Vector Lab, Burlingame, USA). Slides were observed and imaged using a Zeiss Axioplan 2 fluorescence microscope (Zeiss, Oberkochen, Germany). Primary and secondary antibodies used are listed in Table 1.

### Enzyme-linked immunosorbent assay (ELISA)

MCP-1 protein in the culture media was quantified by ELISA (R&D system) following the manufacturer’s instruction. Briefly, 200 μl of standards and culture medium were added to microplates coated with MCP-1 antibody and incubated for 2 h at room temperature. Solution from each well was aspirated and 400 μl of washing buffer added. The wash step was repeated two more times. MCP-1 conjugates (200 μl) were added to each well and incubated for 1 h at room temperature. Subsequently, the microplates were washed three times with washing buffer, incubated with substrate solution (200 μl/well) for 30 min followed by the stop solution (50 μl/well). The optical density of each well was determined by Tecan GENios Pro microplate reader at 450 nm. Actual MCP-1 concentrations were determined using the standard curve generated with MCP-1 standards of known concentrations.

### Real time quantification PCR

Real time PCR was performed as described previously according to the manufacturer’s instructions [24]. In summary, total RNA was extracted from HCE-T cells using the RNeasy Mini Kit (Qiagen, Hilden Germany) and RNA concentration determined by the nanodrop method [25]. One microgram of RNA for each condition was used to synthesize cDNA. First strand synthesis was performed using Superscript II Reverse Transcriptase (Life Technologies, CA, USA) and real time PCR reaction was performed with Roche UPL Mastermix and an appropriate probe from the human library (Roche Applied Science, Penzburg, Germany). Forward and reverse primers used are: 5’TTCTGTGCCTGCTGCTCAT3’ and 5’GGGGCATTGATTGCATCT3’ respectively. GAPDH was used as the endogenous control, forward and reverse primers are 5’AGCCACATCGCTGAGACA3’ and 5’GCCCAATACGACCAAATCC3’ respectively. PCR cycles were performed on Roche LightCycler 480 (Roche Diagnostics, Basel, Switzerland) with the following conditions: denaturation at 95 °C for 10 min, followed by 24 cycles of denaturation at 95 °C for 10 s, annealing at 54 °C for 10 s and extension at 72 °C for 30 s. Delta-Delta Ct method was used to analyse data and fold change was expressed relative to GAPDH levels.

### Statistical analysis

In the NF-кB activity assay, we performed the cell cultures in 3 independent wells and repeated 3 further wells on a different occasion. From each of the wells, the culture media were removed and split into 4 replicates for evaluation of the luminescence. The mean of the 4 replicates (which originated from a single well of cells) was evaluated. The means were used to calculate overall mean of the wells that had the same experimental treatment. To demonstrate overall findings in a graphical format, the means over the 2 occasions (which had 6 independent wells) were then calculated. The same calculation method was used for MCP-1 real time PCR and ELISA assay. All results are expressed as the mean ± standard error of the mean. Statistical analyses were performed using student’s t-test for statistical comparison between different conditions. *P* values less than 0.05 were considered statistically significant.

## Results

### NF-κB was activated through TLR2 in human corneal epithelial cells

The NF-κB activity of HCE-T cells stimulated with Pam3CSK4 for 6 and 24 h was compared to the un-stimulated cells using SEAP assay. At both 6 and 24 h, Pam3CSK4 treated HCE-T cells showed significantly higher levels of NF-κB activity compared to controls (Fig. 1a, column 3 compared to columns 1 and 2). Protein level of NF-κB active subunit p50 increased in the nuclear fractions of Pam3CSK4 treated cells as early as 1.5 h (Fig. 1b). At the same time, cytosolic p50 and p65 decreased over time (data not shown). Immunostaining of the HCE-T cells treated with Pam3CSK4 over the time intervals 0, 1, 2 and 4 h showed increase of p50 and p65 in the nuclei (data for 4 h shown in Fig. 1c). There was an increase of phospho-IκB-α overtime with Pam3CSK4 treatment, while the level of total IκB-α protein remained relatively constant (Fig. 1d).

### Pam3CSK4-stimulated NF-κB activation was mediated by IκK

To investigate whether Pam3CSK4-stimulated NF-κB activation is mediated by IκK, IκK inhibitors Wedelolactone and BMS-345541 were added to cells 1 h before stimulating cells with Pam3CSK4. SEAP assay showed that both Wedelolactone and BMS-345541 could significantly inhibit Pam3CSK4-induced NF-κB activity (Fig. 1a, column 3 compared to columns 5 and 7). Pam3CSK4-induced phosphorylation of IκB-α, the inhibitory subunit of NF-κB, was also suppressed by these inhibitors (Fig. 1d), indicating that Pam3CSK4 stimulated NF-κB activity was mediated by IκK.

### MCP-1 was up-regulated by TLR2-NF-κB activation

**Fig. 2:**
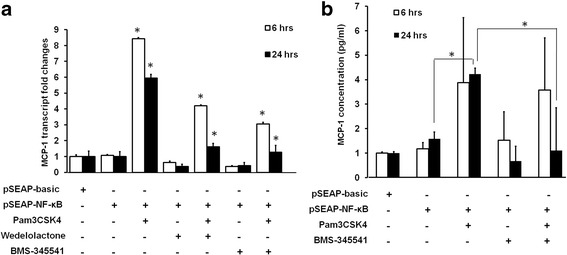
MCP-1 transcript and protein expression in HCE-T cells. **a** qPCR results of MCP-1 transcript levels in control HCE-T cells, and in HCE-T cells transfected with pSEAP-basic or pSEAP-NF-κB, either stimulated or un-stimulated with Pam3CSK4, treated or un-treated with IκK inhibitors, Wedelolactone and BMS-345541. GAPDH was used as the housekeeping control. **b** MCP-1 protein concentration in various cell culture media. Cell culture media were collected from cells transfected with pSEAP-basic or pSEAP-NF-κB with various treatment. ELISA was used to determine MCP-1 protein concentration. The means of two independent experiments are shown. Error bars represent standard error of the means. *: *P* < 0.05

MCP-1 also known as chemokine (C-C motif) ligand 2 (CCL2) is one of the critical regulators for leukocyte recruitment during cornea infection [26, 27]. Transcript levels of MCP-1 were evaluated by qPCR in Pam3CSK4 treated and un-treated cells. Consistent with the NF-κB activity SEAP assay, at both 6 and 24 h, Pam3CSK4-treated HCE-T cells showed higher levels of MCP-1 compared to controls (Fig. 2a, column 3 compared to columns 1 and 2). MCP-1 up-regulation was similarly inhibited with IκK inhibitors Wedelolactone and BMS-345541 (Fig. 2a, column 3 compared to columns 5 and 7). ELISA was used to examine MCP-1 protein levels in the culture media. Results showed that after 24 h treatment, MCP-1 protein in the medium was also up-regulated after Pam3CSK4 stimulation and it was suppressible with BMS-345541 (Fig. 2b) as in the case of the transcript levels.

## Discussion

In this study, we found that in HCE-T cells, NF-κB activity was increased with TLR2 ligand Pam3CSK4 treatment, and the phosphorylation level of IκB-α was also increased with the addition of Pam3CSK4, while total IκB-α protein level remained the same. NF-κB active subunit p50 translocated to the cell nuclei as early as 1.5 h after Pam3CSK4 treatment; NF-κB activation was mediated by IκK. The chemokine MCP-1 was up-regulated by the TLR2 NF-κB pathway at both the transcript and protein levels, and this was also mediated by IκK. Four previous studies evaluated the activation of NF-κB downstream of TLR-2 in cornea epithelial cells. One study investigated NF-κB using the detection of p65 in the nuclear fractions of cells [16], another study investigated NF-κB activity in cell lysate with anti-NF-κB antibodies, whereas two other studies examined the levels of pIκB-α [15, 17]. Neither of these examined the expression of MCP-1 as a possible target, and in addition, the previous studies in corneal epithelial cells did not employ NF-κB promoter assays. In our current report, we examined the activation of NF-κB using a fine tune promoter SEAP assay and reported the regulation of MCP-1 through TLR2 NF-κB pathway. The advantage of using SEAP assay is that the culture medium can be analysed at more than one time point in the experiment without destroying the cells.

Two studies have shown apparently contradictory results when using peptidoglycan (PGN) to treat human corneal epithelial cells, one group found activation of NF-κB as well as increases in IL-6 and IL-8 expressions [17], but not the other [28]. The controversy in this area cannot be easily resolved unless researchers agree to use a common testing strategy for screening potential drugs. We hope that the methodology we propose will be adopted by others and this should help to prevent confusing results from such studies. The strength of this study is the use of more than one approach to investigate the TLR2-NF-κB pathway. The limitation of the study is that in-vivo animal models of Gram-positive infection was not investigated and compared to our in-vitro findings.

In many experimental scenarios, it is valuable to screen a variety of potential new therapeutic agents that may treat Gram-positive eye infections. It is too expensive and resource intensive to investigate all these agents in animal studies in the first instance. It is therefore advantageous to be able to rapidly investigate NF-κB agents, especially those suspected to act via the IκK kinases, in a cell culture system such as the one in our current study to shortlist more promising candidates and reduce the number of molecules that progress to further evaluation in animal models.

## Conclusions

In conclusion, the NF-κB pathway can be activated by the TLR2 ligand and inhibited by IκK inhibitors in the ocular surface cell culture system. This cell culture system may be used to evaluate TLR-related innate defences in ocular surface diseases.
